# Maculosin, a non-toxic antioxidant compound isolated from *Streptomyces* sp. KTM18 

**DOI:** 10.1080/13880209.2021.1946091

**Published:** 2021-07-08

**Authors:** Babita Paudel, Rukusha Maharjan, Prajwal Rajbhandari, Niraj Aryal, Saefuddin Aziz, Keshab Bhattarai, Bikash Baral, Rajani Malla, Hari Datta Bhattarai

**Affiliations:** a Central Department of Biotechnology, Tribhuvan University, Kirtipur, Nepal; b Department of Applied Microbiology and Food Technology, Research Institute for Bioscience and Biotechnology (RIBB), Kathmandu, Nepal; c Department of Pharmaceutical Biology, Eberhard Karls University, Tübingen, Germany; d Department of Biochemistry, University of Turku, Turku, Finland; e Central Department of Botany, Tribhuvan University, Kirtipur, Kathmandu, Nepal

**Keywords:** Bioactivity, brine shrimp toxicity, chromatography, diketopiperazine, DPPH free radical, natural products

## Abstract

**Context:**

*Streptomyces* species are prolific sources of bioactive secondary metabolites known especially for their antimicrobial and anticancer activities.

**Objective:**

This study sought to isolate and characterize antioxidant molecules biosynthesized by *Streptomyces* sp. KTM18. The antioxidant potential of an isolated compound and its toxicity were accessed.

**Materials and methods:**

The compound was purified using bioassay-guided chromatography techniques. Nuclear magnetic resonance (NMR) experiments were carried out for structure elucidation. The antioxidant potential of the isolated compound was determined using DPPH free radical scavenging assay. The toxicity of the isolated compound was measured using a brine shrimp lethality (BSL) assay.

**Results:**

Ethyl acetate extract of *Streptomyces* sp. KTM18 showed more than 90% inhibition of DPPH free radical at 50 µg/mL of the test concentration. These data were the strongest among 13 *Streptomyces* isolates (KTM12–KTM24). The active molecule was isolated and characterized as maculosin (molecular formula, C_14_H_16_N_2_O_3_ as determined by the [M + H]^+^ peak at 261.1259). The DPPH free radical scavenging activity of pure maculosin was higher (IC_50_, 2.16 ± 0.05 µg/mL) than that of commercial butylated hydroxyanisole (BHA) (IC_50_, 4.8 ± 0.05 µg/mL). No toxicity was observed for maculosin (LD_50_, <128 µg/mL) in brine shrimp lethality assay (BSLA) up to the compound’s antioxidant activity (IC_50_) concentration range. The commercial standard, berberine chloride, showed toxicity in BSLA with an LD_50_ value of 8.63 ± 0.15 µg/mL.

**Conclusions:**

Maculosin may be a leading drug candidate in various cosmetic and therapeutic applications owing to its strong antioxidant and non-toxic properties.

## Introduction

Reaction oxygen species (ROS) are generated due to oxidation reactions within living organisms. These ROS are responsible for various degenerative diseases such as deoxygenation of ischaemic tissues, premature ageing, atherosclerosis and cancer (Halliwell and Gutteridge 1990), cardiovascular diseases (Kris-Etherton et al. 2002), neurodegenerative diseases (Di Matteo and Esposito 2003) and inflammation (Ames et al. 1993). These ROS attack several biochemical reactions inside the body resulting in various biochemical disorders (Dean et al. 1993). Stressful environmental conditions and ageing are directly associated with increased ROS production in the human body. Antioxidants can help treat such ROS-induced disorders by terminating chain reactions by being oxidized themselves (Totour 1990).

*Streptomyces* are Gram-positive bacteria that are found in various environmental conditions and have a filamentous mycelium similar to fungi. Phylogenetically, *Streptomyces* is a part of Actinobacteria, with high GC-rich (70%) content. Most of them are ubiquitous and highly versatile soil-dwelling saprophytes known to produce diverse secondary metabolites, many of which are well-known antibiotics (Omura et al. 2001; Khan et al. 2011). Antioxidant activities of 30 strains of rare Actinomycetes were reported (Mohammadipanah and Momenilandi 2018) without characterizing the active molecules. Similarly, the broth extract of *Streptomyces carpaticus* displayed a significant DPPH free radical scavenging activity (IC_50_, 84.5 µg/mL) (Subramanian et al. 2017); however, the active molecule was not characterized. Thus, besides antibiotics, *Streptomyces* could be a promising source of antioxidant compounds.

Here, we have presented the strong antioxidant activity of *Streptomyces* sp. KTM18 and its active antioxidant molecule, maculosin.

## Materials and methods

### Bacteria isolation and maintenance

A total of 20 different soil samples from the depth of 15 cm below the surface were harvested in March 2017 from the Bagmati riverbank of Kathmandu valley (latitude: 27° 39′ 5.39″N; longitude: 85° 17′ 13.80″E). Bacterial colonies were raised at 28 °C on the solid surface of the Yeast Extract Malt Extract (ISP2) agar medium (pH 7.2) by using serially diluted (10^−1^ to 10^−6^) soil samples in sterile water. The seven days grown bacterial colonies were purified by repeated streaking on the solid ISP2 medium. The biochemical properties – Gram-staining, carbohydrates, catalase and starch utilization tests of isolated colonies were performed using agar plate assay.

### Broth culture and extraction

A small scale (3 mL) bioreactor was used to culture all the strains in the ISP2 broth medium at 28 °C at 150 rpm for seven days to screen the antioxidant potential of the isolates. The culture broth was directly freeze-dried, extracted with methanol, and was dried in a vacuum.

### DPPH free radical scavenging assay

The free radical scavenging activity of the test sample was estimated based on the previously described method (Blois 1958) with some modifications. Briefly, 1 mL of DPPH solution (0.1 mM of DPPH in methanol) was mixed with 3 mL of various concentrations of the test sample (0–128 µg/mL). The mixture was incubated at room temperature for 30 min, and the absorbance was measured at 517 nm in a UV-Visible spectrophotometer (Shimadzu UV2600i, Kyoto, Japan). Reaction mixtures without the test sample and with BHA were used as negative and positive controls, respectively. The experiments were conducted in three biological replicates. IC_50_ was calculated by analysing the linear regression of the obtained data.

### Brine shrimp lethality test

Brine shrimp lethality test (BSLT) was used to evaluate the toxicity of test samples as described previously (Meyer et al. 1982). The eggs of *Artemia salina* were hatched in aerated artificial seawater in natural light at 25 °C. The hatched active larvae attracted to light were collected for their toxicity assessment. The active larvae (*n* = 100) were treated with various concentrations of maculosin (0–128 μg/mL). The test samples’ effect was monitored by the manual inspection of live larvae after 24 h of treatment. The mortality rate of the larvae indicated the toxicity of the test samples. Berberine hydrochloride, a standard anticancer drug, was taken as a positive control, and brine shrimp larvae in the artificial seawater were taken as a negative control.

### Metabolic profiling of isolated bioactive compound

A large-scale fermentation of *Streptomyces* sp. KTM18 displaying the antioxidant properties was performed in 10 L volume of ISP2 broth medium (pH 7.2) at 28 °C at 150 rpm for eight days. The mycelial pellets were separated by centrifuging at 5000×*g*, 4 °C for 10 min. The supernatant was extracted three-times with the double volume of butanol (Fisher, Waltham, MA). The organic phase was evaporated at 40 °C in a vacuum using a rotary evaporator (BUCHI, Flawil, Switzerland). The filtered mycelia were extracted with methanol (Fisher, Waltham, MA). The TLC profiles (at 20% methanol in dichloromethane) of both supernatant and mycelial extracts were found almost identical, and both extracts were pooled together (6.5 g total mass). DPPH based antioxidant bioassay-guided fractionation of the crude extract by various chromatography techniques such as Sephadex LH20, silica gel preparative thin-layer chromatography (PTLC) and high-performance liquid chromatography (HPLC) until a pure active compound **1** was obtained. High-resolution mass spectrometry (HR-ESIMS) and nuclear magnetic resonance (NMR) experimental data were used to elucidate the molecular structure of the isolated compound.

### Identification of antioxidant compound producing bacteria

The most antioxidant activity showing strain, KTM18, was identified using 16S rRNA sequencing.

## Results

### Isolation of bacterial strains

A total of 30 morphologically different pure colonies (KTM01–KTM30) were isolated. The voucher specimens are deposited under the voucher number 2017SKTM01 to 2017SKTM30 in the microbial culture collection centre of the Research Institute for Bioscience and Biotechnology (Kathmandu, Nepal). All strains showed a positive test in sucrose, glucose, raffinose, galactose and maltose utilization capacity. Catalase and starch utilization and gram staining test were found positive. These characteristics, along with morphological characteristics such as colony texture observed through naked eyes and light microscope, indicated that all the isolates belong to the genus *Streptomyces*.

### Screening of antioxidant active strains

**Figure 1.**
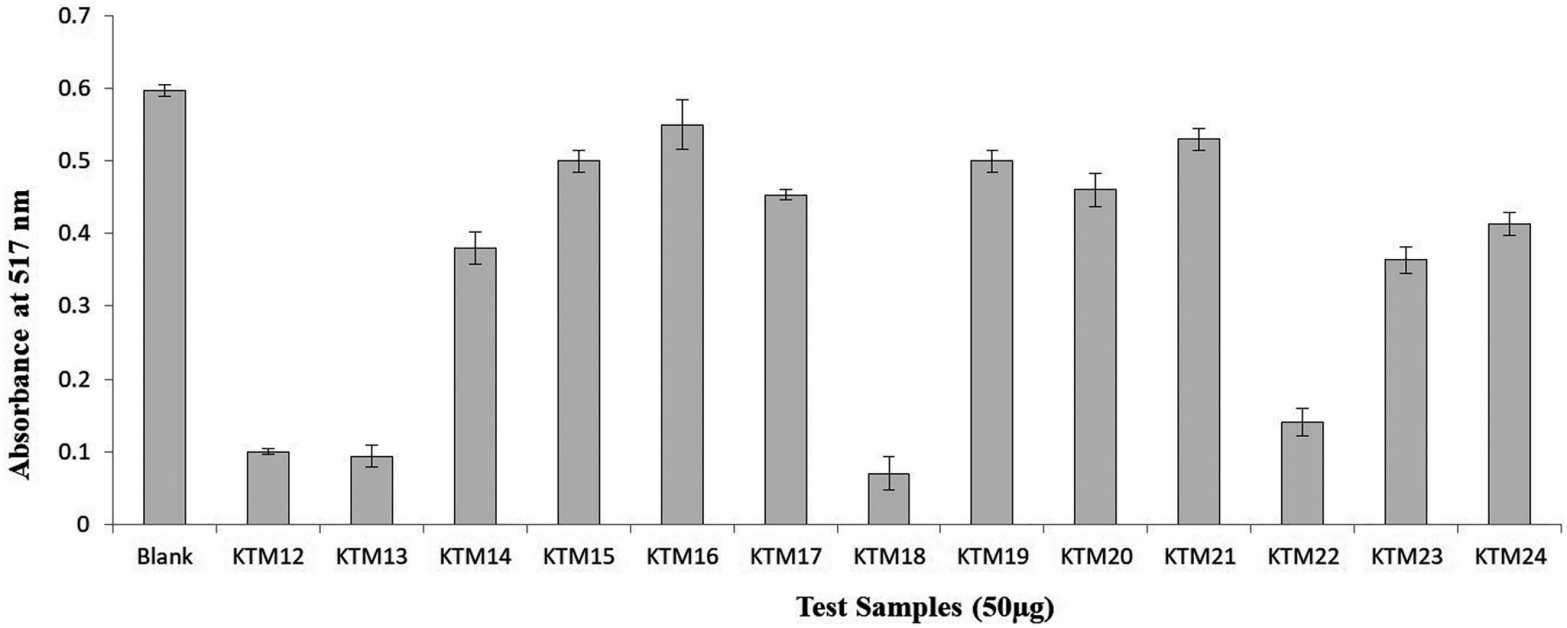
DPPH free radical scavenging activity of seven days old culture broth extract (50 µg) of KTM12–KTM24.

Among 30 isolates, 13 isolates (KTM12–24) showed various degrees of DPPH free radical scavenging activities (10–90%). Among the active isolates, four isolates – KTM12, KTM13, KTM18 and KTM22 revealed comparatively strong DPPH free radical inhibition (75–92%) (Figure 1). Among them, KTM18 displayed the strongest antioxidant activity (92%). Bacterial isolate KTM18 (NCBI accession number, MT517303) was identified as *Streptomyces* sp. by 16S rRNA sequence analysis.

### Identification of maculosin

**Figure 2.**
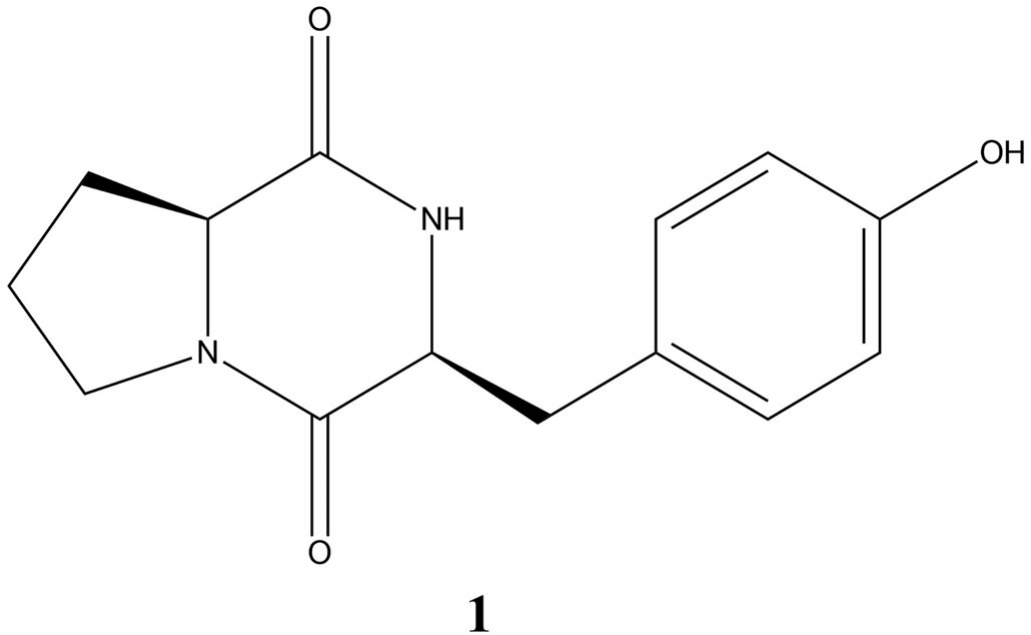
Molecular structure of maculosin.

Compound **1** (Figure 2) was isolated as a pale yellow solid (2.5 mg) with a molecular formula of C_14_H_16_N_2_O_3_ as determined by the [M + H]^+^ peak at 261.1259 of the high-resolution electron spray ionization mass spectrometry (HR-ESIMS) spectrum indicating eight degrees of unsaturation. ^1^H NMR and COSY data (Table 1) revealed a para-substituted benzene ring system. Carbon NMR data showed two ketone carbons, six aromatic carbons, two carbons attached with heteroatoms and four methylene carbons. Cyclo (l-Pro-l-Tyr) structure system was established by using ^1^H NMR, ^13^C NMR and HMBC data.

**Table 1.** NMR data for compound **1** (400 MHz, DMSO-*d*_6_).

Position	*δ*C	C-type	*δ*H (mult. *J* Hz)	HMBC (1H-C)	COSY
1	– (N)	–	–	–	–
2	166.9 (C-2)	C═O	–	–	–
3	57.9 (C-3)	CH	4.36(t, 4.6, H–3)	3, 2.10, 11	–
4	– (N)	–	–	–	–
5	170.7 (C-5)	C═O	–	–	–
6	60.1 (C-6)	CH	4.04 (ddd, 10.8, 6.3, 1.4, H-6)	5, 6, 7, 8	–
7	29.4 (C-7)	CH_2_	2.07–2.12 (1H, m, H-7) 1.19–1.26(1H, m, H-7′)	5, 6, 7, 8	1.81, 4.04
8	22.7 (C-8)	CH_2_	1.78–1.83 (2H, m, H-8)	7, 8, 9	1.23, 3.53
9	45.9 (C-9)	CH_2_	3.51–3.57 (1H, m, H-9), 3.34–3.38 (1H, m, H-9′)	2, 6, 7, 8, 9	1.81
10	37.7 (C-10)	CH_2_	3.09 (1H, dd, 14.2, 4.6, H-10), 3.03 (1H, dd, 14.2, 4.6, H-10′)	3, 2, 10, 11, 12, 16	4.36
11	127.9 (C-11)	Cq	–	–	–
12	132.4 (C-12)	CH	7.01(d, 8.4, H-12)	3, 10, 12, 13, 14, 15, 16	6.72
13	116.2 (C-13)	CH	6.72(d, 8.4, H-13)	11, 13, 14, 15	7.01
14	157.7 (C-14)	C-OH	–	–	–
15	116.2 (C-15)	CH	6.72(d, 8.4, H-15)	11, 13, 14, 15	7.01
16	132.4 (C-16)	CH	7.01(d, 8.4, H-16)	3, 10, 12, 13, 14, 15, 16	6.72

### Antioxidant and brine shrimp toxicity of maculosin

Maculosin revealed DPPH free radical scavenging activity stronger than the commercially used butylated hydroxyanisole (BHA) (Table 2). In addition, it showed no toxicity in brine shrimp lethality assay (BSLA) up to the test concentration of 128 µg/mL. The commercial standard, berberine chloride, displayed 50% death (LD_50_) of test larvae at 8.63 ± 0.55 µg/mL of the test concentration.

**Table 2.** Antioxidant activity and brine shrimp toxicity activity of maculosin.

Test samples	DPPH free radical scavenging activity (50% inhibition, IC_50_) (µg/mL)	Brine shrimp lethality test (50% lethal dose, LD_50_) (µg/mL)	Remark
Maculosin	2.16 ± 0.05	NA^a^	
BHA	4.8 ± 0.15	–	+ve control
Berberine chloride	–	8.63 ± 0.55	+ve control

NA: no activity.

^a^ Activity was not observed until the test concentration of 128 µg/mL.

## Discussion

A total of 30 colonies (KTM1–KTM30) were isolated with a distinct morphological characteristic based on colony morphology, colour and texture. Based on colony phenotypic characteristics and biochemical tests (Gram staining, positive carbohydrate utilization test, positive starch hydrolysis test and positive catalase tests), all the isolates were primarily identified as *Streptomyces* spp. Based on the DPPH free radical scavenging assay of the crude extract (50 µg) of isolates, 13 isolates (KTM12–KTM24) were screened further and are presented in this manuscript. Among these 13 isolates, four isolates – KTM12, KTM13, KTM18 and KTM22 displayed 75–90% scavenging of DPPH free radicals. The crude extract of KTM18 showed the strongest antioxidant activity (90% inhibition). Therefore, KTM18 was subjected further to large-scale fermentation and bioassay-guided isolation leading to the characterization of the bioactive molecule.

DPPH free radical scavenging activity-based bioassay-guided fractionation using various chromatography experiments and characterization of isolated molecules using NMR experiments yielded maculosin. The NMR data (Table 1) obtained for compound **1** were comparable with cyclo (L-Pro-L-Tyr), as described previously (Wattana-Amorn et al. 2016). The absolute stereochemistry of **1** was established by comparing the ^1^H NMR spectrum of compound **1** with synthetic cyclo (L-Pro-L-Tyr) (Wattana-Amorn et al. 2016). The NMR spectra of compound **1** are presented in the Supplementary material. Besides, the maculosin isolated in this research was not a media artefact. The LC-ESIMS profile of the ISP2 broth medium did not detect the ion peak of maculosin.

In the present research, maculosin showed stronger antioxidant activity than the commercially used BHA (Table 2). Previously, maculosin was described as host-specific phytotoxin isolated from *Alternaria alternata* on *Centuria maculosa* (Stierle et al. 1988). Similarly, maculosin isolated from *Pseudomonas rhizosphaerae* was reported as an antibacterial agent against various marine bacteria, including *Ruegeria* sp., *Bacillus cereus* and *Pseudoalteromonas piscida* (Qi et al. 2009). A glycoside of maculosin isolated from marine *Streptomyces* sp. ZZ446 showed strong antimicrobial activity (26–37 µg/mL) against methicillin-resistant *Staphylococcus aureus*, *Escherichia coli* and *Candida albicans* (Chen et al. 2020). DPPH based antioxidant activity of maculosin was reported here for the first time from the *Streptomyces* sp. This is in congruence with our current findings where no toxicity of maculosin was observed in the BSLA until the test concentration of 128 µg/mL. However, available literature indicated that maculosin showed cytotoxic activity (IC_50_, 48.90 µg/mL) against the human liver cancer cell lines (Karanam et al. 2020). Such observations indicated that maculosin’s cytotoxic activity is selective against the liver cancer cell line. Industrial antioxidants have diverse uses, such as medicines, food and cosmetics preservatives, and inhibitors of rubber or gasoline deterioration. Taken together, the antioxidant, anti-cancerous and non-toxicity properties displayed by the maculosin suggested that the compound possessed a high therapeutic value and could act as a lead drug candidate in the future.

## Conclusions

The present study investigated the strong antioxidant activity of maculosin when compared to the commercial antioxidant compound, BHA. Furthermore, maculosin was found to be non-toxic against brine shrimp larvae. However, further research on antioxidant mechanisms and detailed investigation on the toxicity of maculosin on various human cell lines seems essential to provide a further scientific basis regarding the potential of this natural molecule. Based on these biological activities, maculosin could be a potent therapeutic or cosmetic drug candidate for further research.

## Supplementary Material

Supporting_Info-maculosin-final.docx
