# Supplementary material for: Maculosin, a non-toxic antioxidant compound isolated from Streptomyces sp. KTM18
Source: Pharm Biol. 2021 Jul 8;59(1):931–4. doi: 10.1080/13880209.2021.1946091 (PMC8274501; doi:10.1080/13880209.2021.1946091)
Supplement: Supporting_Info-maculosin-final.docx [file IPHB_A_1946091_SM7884.docx]

**Maculosin, a nontoxic antioxidant compound isolated from *Streptomyces* sp. Strain KTM18**

Babita Paudel^1^, Rukusha Maharjan^2^, Prajjwal Rajbhandari^2^, Niraj Aryal^3^, Saefuddin Aziz ^3^, Keshab Bhattarai^3^, Bikash Baral^4^, Rajani Malla^2^, and Hari Datta Bhattarai^3, 5*^

^1^Central Department of Biotechnology, Tribhuvan University, Kirtipur, Kathmandu, Nepal

^2^Department of Industrial Microbiology, Research Institute for Bioscience and Biotechnology (RIBB), Kathmandu, Nepal

^3^Department of Pharmaceutical Biology, Eberhard Karls University, 72076 Tübingen, Germany

^4^ Department of Biochemistry, University of Turku, Finland

^5^Central Department of Botany, Tribhuvan University, Kirtipur, Kathmandu, Nepal

*Address for correspondence, H. D. Bhattarai, [haridatta.bhattarai@cdb.tu.edu.np](mailto:haridatta.bhattarai@cdb.tu.edu.np)

***Supporting Information (SI)***

Table of Contents

Figure S1. DPPH free radical scavenging activities of 100 µL of 7 day old culture broth of five active strains. The change of purple color of DPPH into yellow indicates the DPPH scavenging activity.

Figure S2. 1H NMR spectrum of maculosin (400 MHz, DMSO-d_6_).

Figure S3. 13C NMR spectrum of maculosin (100 MHz, DMSO-d_6_)

Figure S4. COSY spectrum of maculosin.

Figure S5. HSQC spectrum of maculosin.

Figure S6. HMBC spectrum of maculosin.

Figure S7. HR-ESIMS spectrum of maculosin.

Figure S8. Maximum UV absorbance ( λ_max_) spectrum of maculosin..

Figure S7. HPLC spectrum of isolated pure maculosin.

**Physiological and biochemical characterization of isolates**

**Gram staining**

Gram-staining of isolates was done using HIMEDIA Gram Stains-Kit. A smear of the selected strains was prepared on a clean glass slide and allowed to air-dry. The smear on the slide was fixed by passing through the Bunsen burner and then flooded with crystal violet. After one min, it was rinsed with D/W and flooded with mordant Gram’s iodine. After again rinsing with D/W the smear was decolorized with ethanol, washed with D/W, and counter-stained with safranin for 45 s. Then the smear was rinsed for the last time and allowed to air dry. It was then examined under a light microscope.

**Catalase test**

1-2 drops of culture broth of each strain were placed on a glass slide and 1-2 drops of H_2_O_2_ were added on the slide. Results were recorded after 15 s. The release of oxygen gas bubbles was recorded as the positive catalase test result.

**Starch hydrolysis test**

Isolates were inoculated on starch agar plates (beef extract 3.0 g/L, soluble starch 10.0 g/L, agar 15 g/L, pH 7.6) for 72 h at 28 ^○^C. After incubation, the surfaces of the agar plates were flooded with Gram’s iodine solution and the results were observed after 1-2 min. The appearance of a clear halo around the colonies indicated a positive result due to the production of the amylase enzyme by the tested isolates. The absence of the clear halo around the colonies indicated as a negative starch hydrolysis test.

**Carbohydrates utilization test**

Phenol red broth (100 mL for each test) was prepared using nutrient broth media and 1 µg/mL of phenol red. After autoclaving the media, 1% carbohydrate was mixed in the phenol red broth media and the final pH was maintained around 7.5-8. Sucrose, glucose, maltose, raffinose, and galactose were the five chosen carbohydrates for our test. Change in color of media from light red to yellow was taken as a positive result whereas no change indicated any fermentation of sugar.

**Nucleic acid extraction, 16S rRNA gene amplification, and sequencing:**

Bacterial culture broth of 5 ml was centrifuged (3 min, 5,000 × *g*, 4^○^C), washed with STE buﬀer, and treated with 1.4 M NaCl, 20 mM EDTA, 100 mM Tris-HCl (pH 8.0), 5 mg/mL proteinase K, 2 mg/mL lysozyme, and 1 mg/mL RNase A. Sample was then frozen at 80^○^C for 3-5 min and thawed in a dry bath for 3 min at 65^○^C (this process was repeated twice). Next, an equal volume of a solution containing phenol/chloroform/isoamyl alcohol 25:24:1 was added to the tube, homogenized by inversion, and centrifuged (10 min, 14,000 × *g*, 4^○^C). The aqueous phase was recovered, mixed with 400 mL of chloroform, and centrifuged (10 min, 14,000 × *g*, 4^○^C). Genomic DNA was precipitated with ammonium acetate and cold isopropyl alcohol and frozen at -20^○^C for 10 min and subsequently centrifuged. The supernatant was discarded, and the pellet formed was washed with cold ethanol (70%), centrifuged, and solubilized in TE buﬀer (10 mM Tris-HCl and 1 mM EDTA). Genomic DNA was quantiﬁed using Biophotometer^TM^. To assess the integrity and purity of the DNA, an agarose gel (1%) was stained with peqGREEN^TM^ and visualized in a transilluminator. The 16S rRNA gene for Nephalis samples was ampliﬁed by polymerase chain reaction (PCR) using primers, 27F (5’-AGAGTTTGATCCTGGCTCAG-3’) and 1492R (5’-TACGGCTACCTTGTTACGACTT-3’) (Gontang et al. 2007). The PCR mix (25 µL) contained DNA (1 µL), primer (0.75 µL), PCR buﬀer (5 µL), DNTPs (1 µL), *Taq* DNA polymerase (0.5 µL) and distilled water (17.5 µL). The thermo cycling conditions included an initial denaturation period at 94^○^C, followed by 35 cycles of denaturation at 94^○^C for 60 s, annealing phase under 63^○^C for 60 s, and extension at 72^○^C for 60 s. This was followed by a ﬁnal extension at 72^○^C for 10 min. The PCR products were visualized in a 1% agarose gel electrophoresis and stained with peqGREEN^TM^. The PCR product puriﬁed by PCR Purification Kit (250) QIAGEN^TM^. The sequencing of the 16S rRNA gene was performed by Eurofins Genomics (Ebersberg, Germany) with the ABI PRISM BigDye^TM^ Terminator cycle sequencing kit (Applied Biosystems, U.S.A), and the same primers were used for ampliﬁcation, following the protocols provided by the manufacturer.

# Table. S1. Biochemical properties of *Streptomyces* strains

| **Isolate**  **name** | **Gram**  **stain** | **Carbohydrate utilization test** | | | | | **Catalase**  **test** | **Starch hydrolysis** |
| --- | --- | --- | --- | --- | --- | --- | --- | --- |
|  |  | **Sucrose** | **Glucose** | **Raffinose** | **Galactose** | **Maltose** |  |  |
| KTM12 | + | + | + | + | + | + | + | + |
| KTM13 | + | + | + | + | + | + | + | + |
| KTM14 | + | + | + | + | + | + | + | + |
| KTM15 | + | + | + | + | + | + | + | + |
| KTM16 | + | + | + | + | + | + | + | + |
| KTM17 | + | + | + | + | + | + | + | + |
| KTM18 | + | + | + | + | + | + | + | + |
| KTM19 | + | + | + | + | + | + | + | + |
| KTM20 | + | + | + | + | + | + | + | + |
| KTM21 | + | + | + | + | + | + | + | + |
| KTM22 | + | + | + | + | + | + | + | + |
| KTM23 | + | + | + | + | + | + | + | + |
| KTM24 | + | + | + | + | + | + | + | + |


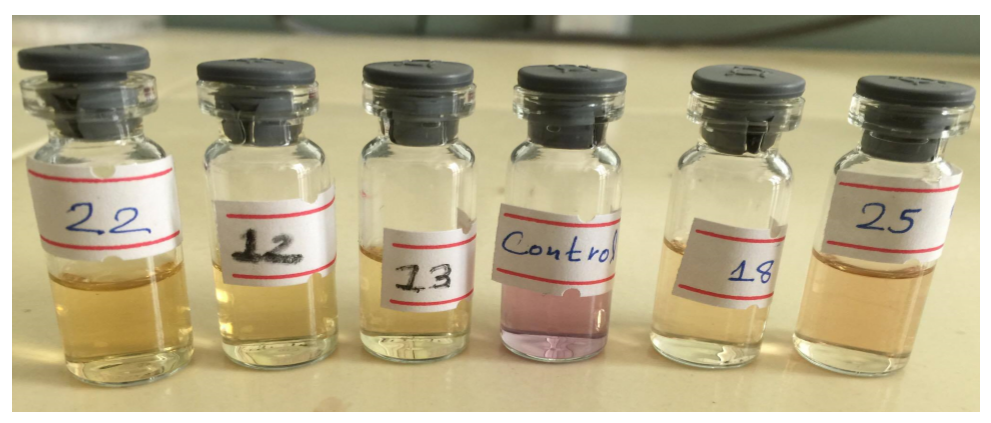


# Figure S1. DPPH free radical scavenging activities of 100 µl 7 day old-cultured broth of five active strains. The change of purple color of DPPH into yellow indicates the DPPH scavenging activity.


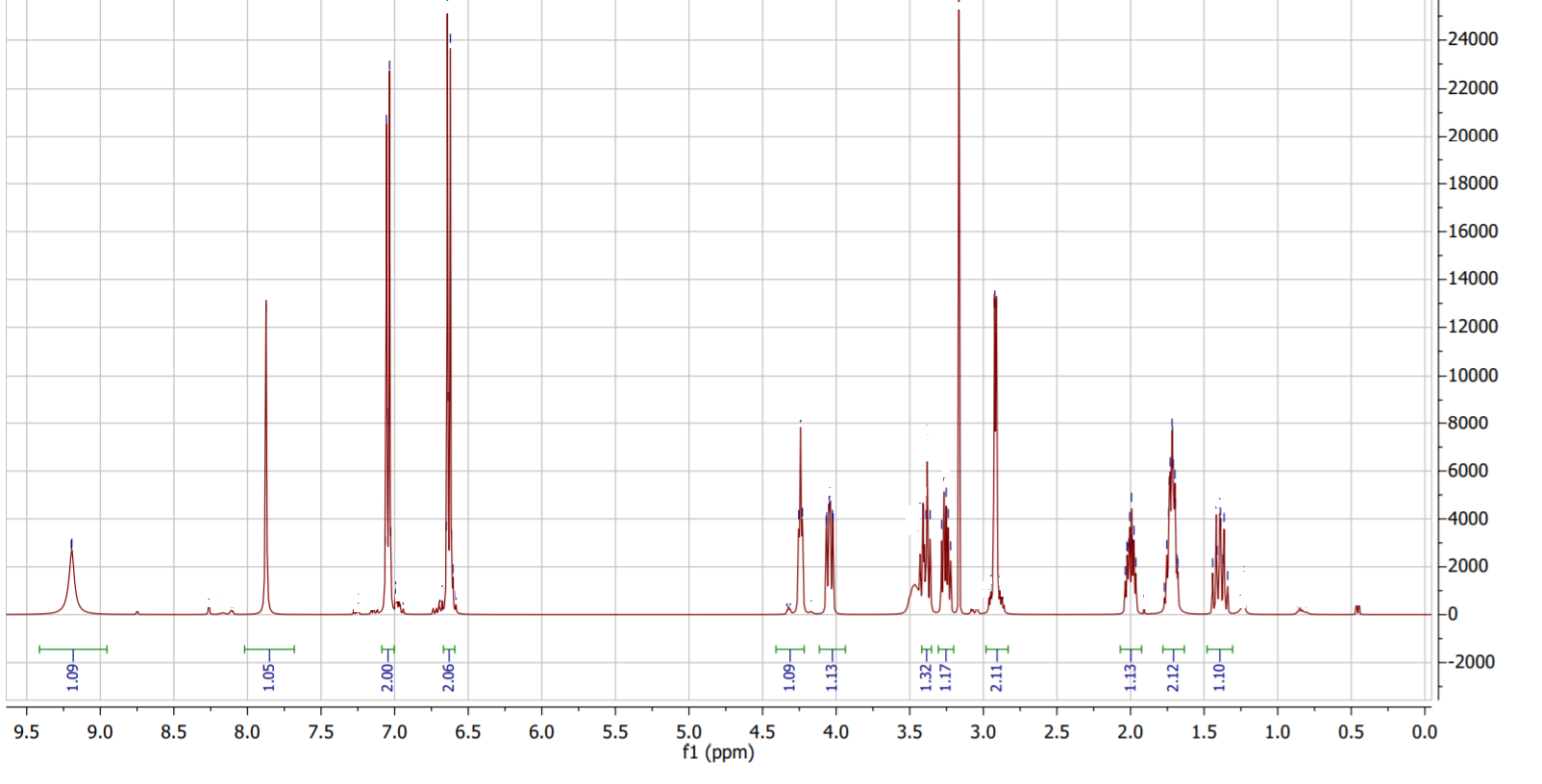


# Figure S2. ^1^H NMR spectrum 400 MHz, DMSO-d_6_.


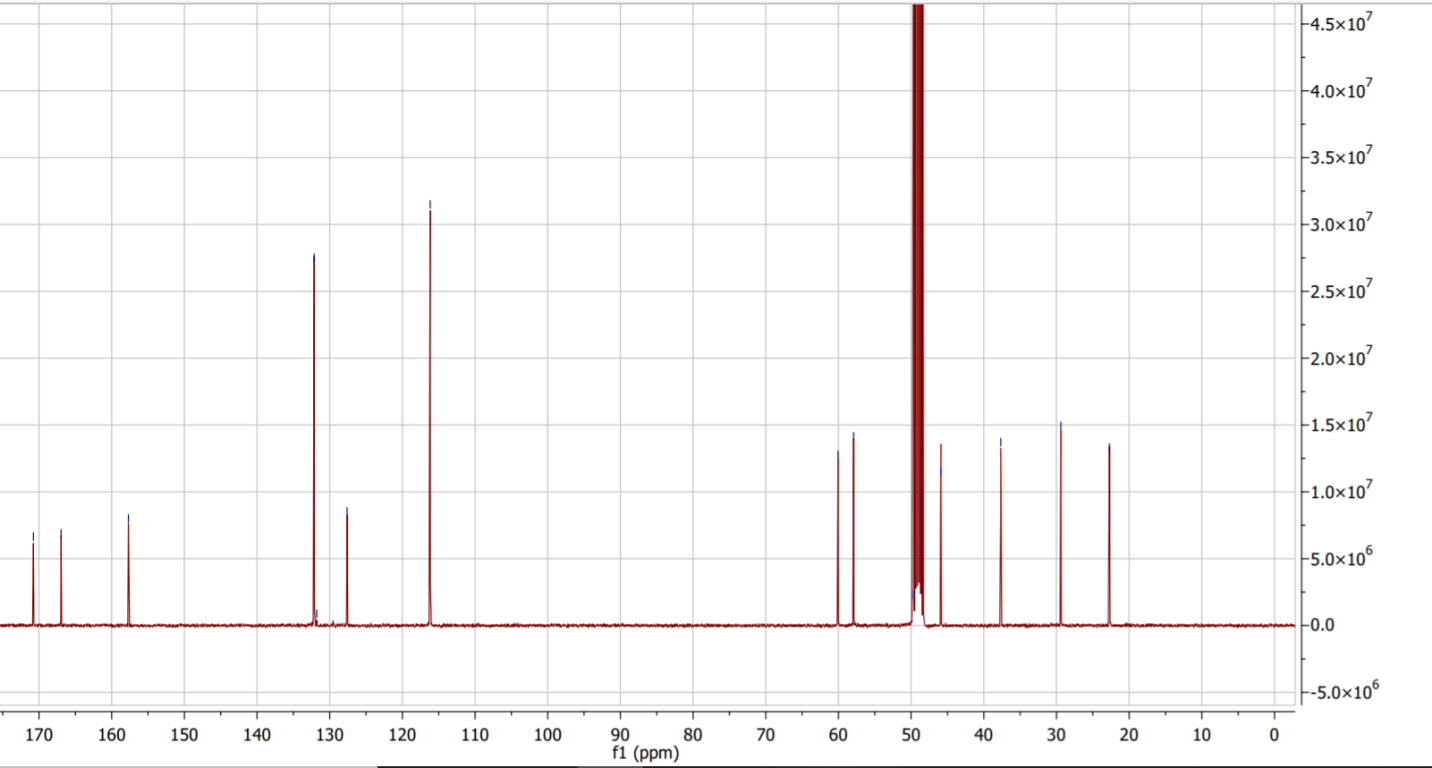


# Figure S3. ^13^C NMR at 100 MHz, DMSO-d_6_.


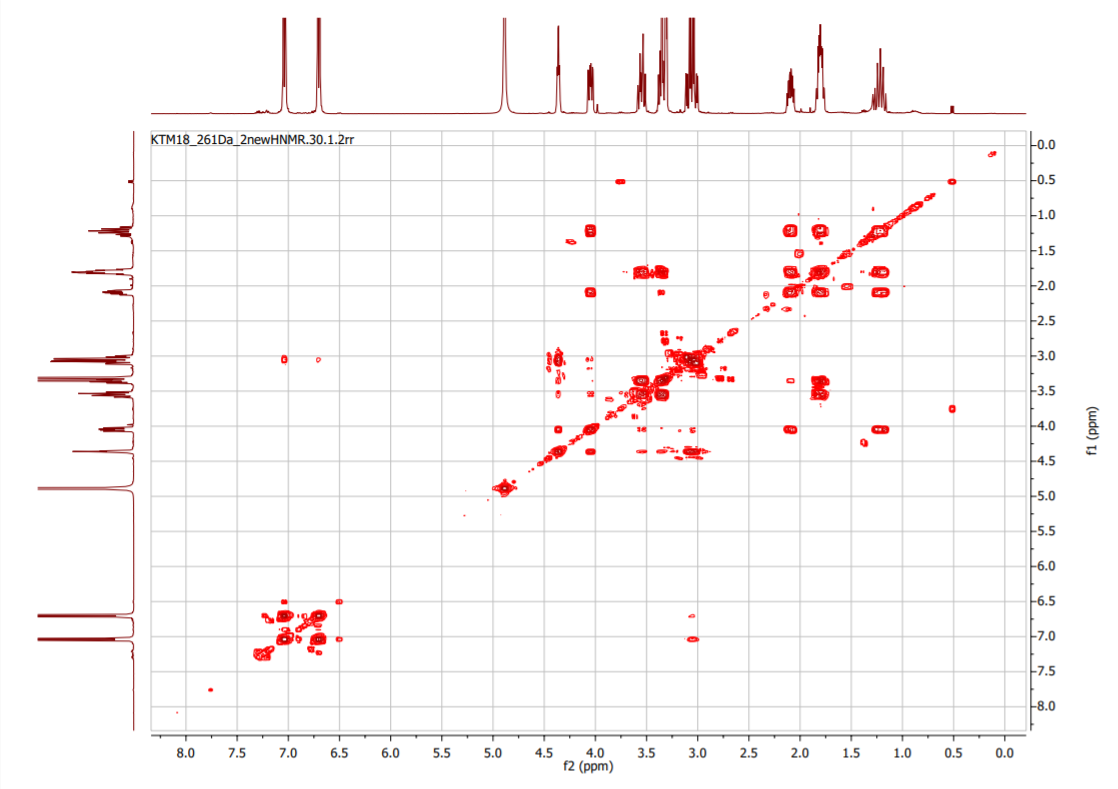


# Figure S4. COSY Spectrum of maculosin.


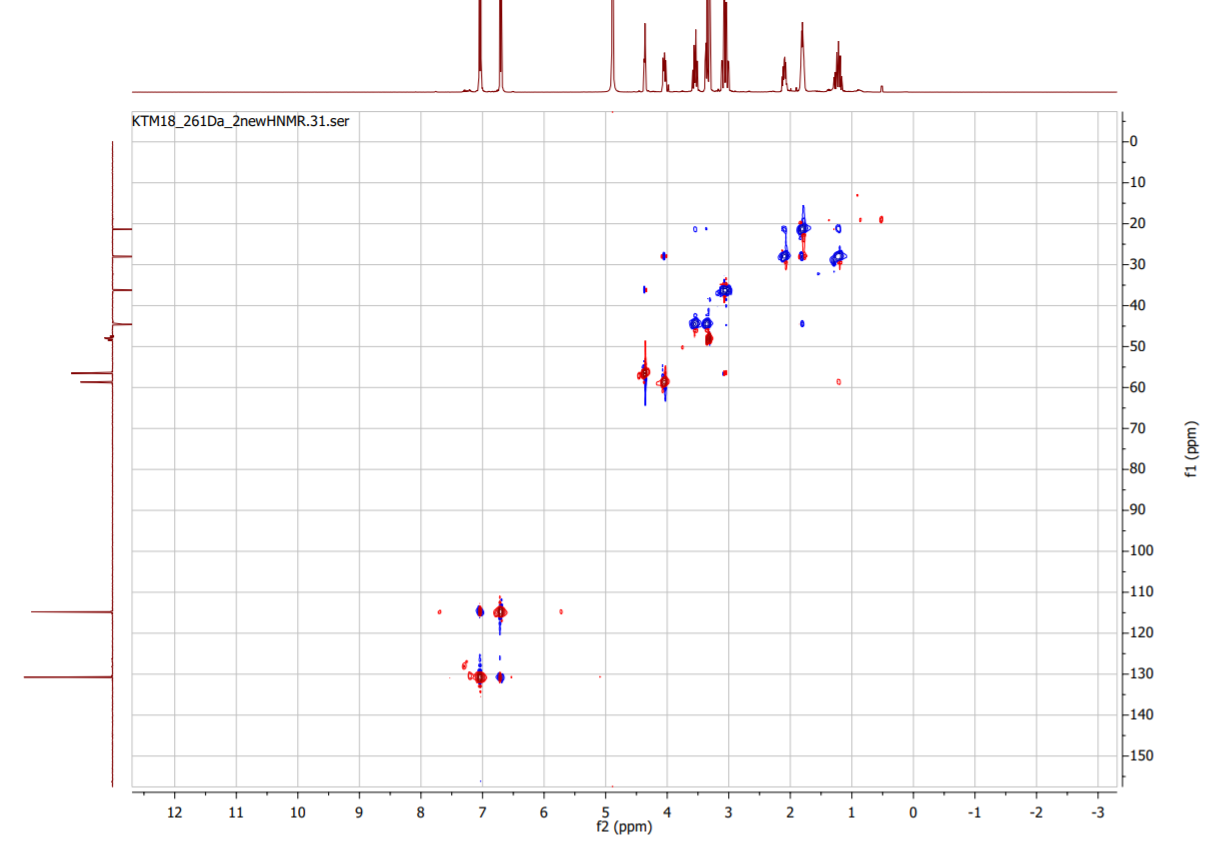


# Figure S5. HSQC Spectrum of maculosin.


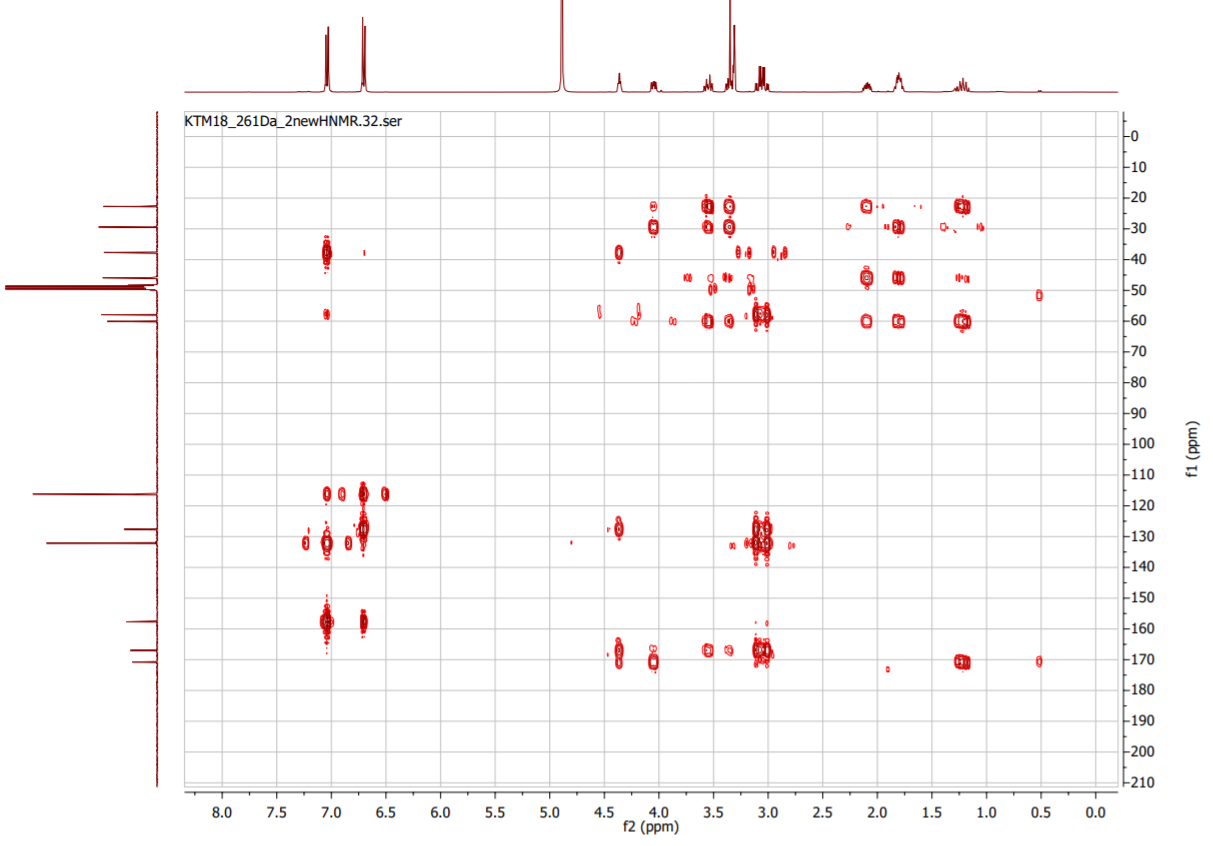


# Figure S6. HMBC spectrum of maculosin.

**Figure S7**. HR-ESIMS spectrum of maculosin.

**Figure S8**. Maximum UV absorbance ( λ_max_) spectrum of maculosin.

**Figure S9**. HPLC spectrum of isolated pure maculosin.

**References:**

Gontang EA, Fenical W, Jensen PR. 2007. Phylogenetic diversity of gram-positive bacteria cultured from marine sediments. Appl Environ Microbiol. 73:3272-3282.
